# Validation and Acceptability of the Mobile App Version of the Control of Allergic Rhinitis and Asthma Test for Children (CARATKids): Cross-Sectional Study

**DOI:** 10.2196/73531

**Published:** 2025-07-31

**Authors:** Dulce Abreu da Mata, Inês Pais-Cunha, Sandra Catarina Ferraz, Daniela da Rocha Couto, Catarina Ferraz, Sónia Silva, José Carlos Valente, Pedro Vieira-Marques, João A Fonseca, Inês Azevedo, Cristina Jácome

**Affiliations:** 1 Unidade Autónoma de Gestão da Mulher e Criança, Pediatrics Department, Unidade Local de Saúde São João, Porto, Portugal; 2 Pediatrics Department, Unidade Local de Saúde do Alto Minho, Viana do Castelo, Portugal; 3 Pediatrics Department, Unidade Local de Saúde da Cova da Beira, Covilhã, Portugal; 4 RISE-Health, Departamento de Ginecologia Obstetrícia e Pediatria, Faculdade de Medicina, Universidade do Porto, Porto, Portugal; 5 MEDIDA – Serviços em Medicina, Educação, Investigação, Desenvolvimento e Avaliação, Porto, Portugal; 6 RISE-Health, MEDCIDS- Department of Community Medicine, Information and Health Decision Sciences, Faculty of Medicine, University of Porto, Rua Dr. Plácido da Costa, Porto, 4200-450, Portugal, 351 225513622; 7 Allergy Unit, Hospital CUF Porto, Porto, Portugal

**Keywords:** asthma, rhinitis, allergic, pediatrics, telemedicine, mobile applications, patient-reported outcome measures, psychometrics, reliability, patient preference, mobile phone

## Abstract

**Background:**

The electronic version of the Control of Allergic Rhinitis and Asthma Test for Children (CARATKids) has the potential to enhance pediatric telemonitoring but has not yet been validated.

**Objective:**

This study aimed to validate the electronic version of CARATKids against the paper-based version.

**Methods:**

A cross-sectional study was conducted between April and December 2024 in a tertiary hospital in northern Portugal. Children with asthma or allergic rhinitis and their caregivers were recruited during pulmonology outpatient appointments. CARATKids comprises 13 yes or no questions, 8 addressed to the child and 5 to the caregiver, and the total score ranges from 0 to 13. The electronic CARATKids was made available through a mobile app. Both paper and electronic versions were administered in a randomized order before and after the appointment. In addition, participants’ preferences between the two administration versions were assessed. Internal consistency (Cronbach α), reliability (intraclass correlation coefficient [ICC], Bland-Altman analysis), and convergent validity (Spearman coefficient) were analyzed following COnsensus-based Standards for the selection of health Measurement INstruments (COSMIN) guidelines.

**Results:**

A total of 51 children (median 9, IQR 8-11 years; n=29, 57% male) and respective caregivers (median 41, IQR 7-45) years were included. The CARATKids total score was similar across the paper (median 5, IQR 3-8) and electronic (median 5, IQR 3-7) versions. The internal consistency was 0.79 for the paper version and 0.83 for the electronic version. The reliability between the two versions was excellent (ICC 0.95, 95% CI 0.91‐0.97). The Bland-Altman analysis showed strong agreement between the two versions, with a mean difference of 0.04 (95% CI −1.99 to 2.07). The Spearman correlation between the two versions was 0.95 (*P*<.001). In total, 63% (n=32) of children and 61% (n=31) of caregivers were indifferent to the version used, while 33% (n=17) and 35% (n=18), respectively, preferred the electronic version.

**Conclusions:**

The electronic version of CARATKids appears to be equivalent to the paper-based version of the questionnaire, with good acceptance by children and caregivers. CARATKids implementation in mobile health technologies has the potential to enhance remote child monitoring and optimize the management of asthma and allergic rhinitis.

## Introduction

Asthma affects 5%‐6% of children worldwide and is a leading cause of disability among those aged 5‐14 years old [1,2]. The condition significantly impacts the quality of life and productivity of both children and their caregivers, leading to substantial costs, with a disproportionate burden on ethnic minority groups and economically disadvantaged populations [3-5]. Allergic rhinitis, another common condition, often develops early in life, with a prevalence of 8.5% at ages 6‐7, rising to 14.6% in those aged 13‐14 years old [6]. The symptoms of allergic rhinitis have a profound negative impact on children’s physical and emotional health, sleep quality, and daily activities [7]. Epidemiological and clinical studies have highlighted the strong link between asthma and allergic rhinitis, leading to the widely recognized concept of United Airways Disease, particularly evident in children [8]. Implementing effective monitoring strategies tailored to individual needs can optimize health outcomes for children with these conditions and their caregivers.

Patient-reported outcome measures (PROM) for asthma and allergic rhinitis assess key aspects of care and provide insights into patients’ perceptions of treatment response over time [9,10]. The Control of Allergic Rhinitis and Asthma Test for Children (CARATKids) is the first questionnaire to simultaneously assess allergic rhinitis and asthma control in children aged 6‐12 years old [11]. It is validated in 7 languages and is a recognized PROM used in clinical practice [11-16]. While the CARATKids paper version remains a valuable tool during scheduled in-person appointments, it often provides limited insight into the child’s health trajectory in the period in-between appointments, as it relies on a recall period of 2 weeks. In this context, electronic patient-reported outcome measures (ePROMs) offer a complementary solution by enabling continuous long-term tracking and monitoring of symptoms, allowing health professionals to make more informed decisions based on real-life patient data [17-19]. This approach facilitates a more proactive and personalized approach to child care, allowing health professionals to identify trends and changes in a patient’s condition over time [17,18,20]. In addition, ePROMs can offer numerous advantages, including greater patient acceptability, enhanced data quality, and higher response rates, while providing additional benefits for health systems and research [17,18,20,21].

The Allergic Rhinitis and its Impact on Asthma guidelines, implemented in over 70 countries, have evolved to include integrated care pathways with mobile technology for individualized and predictive medicine [22]. There is now an opportunity to increase access to asthma and allergic rhinitis care, ensure better disease control, bridge equity gaps, and control costs. A mobile app version of CARATKids could play a key role in integrating telemonitoring technologies to support remote management of pediatric asthma and allergic rhinitis.

The adult and adolescent version of the Control of Allergic Rhinitis and Asthma Test (CARAT) questionnaire has already been validated for mobile app use, demonstrating psychometric properties comparable to those of the paper version [23]. To date, CARATKids has only been validated in its paper version [11]. An electronic version of CARATKids should be validated comparing with the traditional paper-based questionnaire before being implemented in a clinical setting [24,25].

The primary objective of this study was to validate the electronic version of CARATKids for children with asthma or allergic rhinitis using a mobile app compared to the paper version. A secondary objective was to investigate the CARATKids preferred version, paper or electronic, by children and their caregivers.

## Methods

### Study Design

This cross-sectional study was conducted at the Pediatrics Department of Hospital de S. João, a public tertiary care hospital in northern Portugal, from April to December 2024. Children and their caregivers were invited to participate during scheduled pediatric pulmonology appointments. This study followed the guidelines on measurement properties for patient-reported outcomes of the COSMIN (COnsensus-based Standards for the selection of health Measurement INstruments) initiative. This study was reported in compliance with the STROBE (Strengthening the Reporting of Observational Studies in Epidemiology) reporting guidelines [26].

### Ethical Considerations

Ethical approval for this study was obtained from the Ethics Committee of São João Local Health Unit (approval 316/20; addendum dated April 19, 2024). Caregivers provided written informed consent, and informed assent was obtained from the children before data collection. Participants had the right to refuse to participate or to withdraw from the study at any time, without penalty or prejudice, and without needing to provide a reason. No financial compensation was provided to participants for their involvement in this study.

### Participants

The sample size was set at a minimum of 50 children and their respective caregivers, following COSMIN guidelines [27]. Participants were selected by physicians based on convenience, specifically on days with a lighter appointment workload and when a researcher was available to assist with data collection. Children aged 6 to 12 years with a scheduled pediatric pulmonology appointment were included if they had a confirmed or suspected diagnosis of asthma or allergic rhinitis. Children were excluded if they had a major respiratory disease other than asthma or allergic rhinitis, or any other medical condition potentially contributing to respiratory symptoms. Caregivers were excluded if they were minors. Both children and caregivers with insufficient knowledge of the Portuguese language or unable to understand or answer self-reported questionnaires were excluded.

### Data Collection

Demographic data from children (sex, age, height, weight, and school year) and their caregivers (sex, age, and level of education) were first collected in a paper case report form. In addition, to estimate the level of digital literacy, both children and caregivers were asked about smart device ownership and whether they had ever downloaded and used a health or fitness app. The CARATKids questionnaire was administered in a randomized order, either as a paper version (pCARATKids) or an electronic version (eCARATKids), with one format completed before and the other after the pediatric pulmonology appointment. Time in-between the two assessments, pCARATKids and eCARATKids, was recorded. Following the administration of both versions, children and caregivers answered about their preference for the paper version, the electronic version, or their indifference between the two.

### CARATKids

The Portuguese original version of CARATKids was used in this study [28]. The questionnaire comprises 13 yes or no questions, with 8 items directed to the child and 5 to the caregiver, referencing a recall period of the previous 2 weeks. Each positive response is assigned a score of 1 point, while a negative response is scored as 0. The total score, which ranges from 0 to 13, is calculated by summing the positive responses. Higher scores indicate poorer control of asthma and allergic rhinitis. Child, caregiver, and total scores were calculated. The CARATKids total score was categorized into 3 levels of disease control: controlled (<4), insufficiently controlled (4 or 5), or uncontrolled (>5) [11]. Throughout the study, participants were not informed of their scores or the corresponding disease control classification to prevent any potential influence on their responses. Participants completed both the pCARATKids (see Multimedia Appendix 1) and eCARATKids with support available from a researcher upon request. The eCARATKids was accessible through the InspirersKids mobile app (MEDIDA), which is available for both iOS- and Android-based devices, and was installed on the researchers’ smartphones. With this mobile app, questions are presented one at a time rather than displaying the entire questionnaire at once (see Multimedia Appendix 2). A pop-up notification alerts users when the child’s section is completed, and the caregiver’s section begins. The app could be used offline (to facilitate its use in the clinical context) and, whenever an internet connection was available, the app automatically transferred the eCARATKids answer files to a secure server at Faculdade de Medicina da Universidade do Porto, from which the files could be accessed only by authorized researchers [29].

### Data Analysis

Descriptive analyses, including the median (IQR) and the absolute and relative frequencies, were performed to characterize the children and their caregivers, as well as to describe CARATKids scores and version preferences.

Given the sample size, the Kolmogorov-Smirnov Test was conducted to assess the normality of the data. If the data followed a normal distribution, parametric methods were applied; otherwise, nonparametric methods were used.

The internal consistency of both pCARATKids and eCARATKids was assessed using Cronbach α. The relative reliability between the two versions was determined using the intraclass correlation coefficient (ICC), using both two-way mixed single measures, while the absolute reliability was evaluated through Bland-Altman plots. Convergent validity between the traditional paper-based version and the mobile app version of CARATKids was assessed through Spearman rank correlation coefficient. According to COSMIN methodology, Cronbach α, ICC, and Spearman correlation coefficients equal to or greater than 0.70 indicate good measurement properties [27].

All statistical analyses were performed using IBM SPSS Statistics (version 29.0.1.0), with the level of significance set at *P*<.05.

## Results

### Participants Characteristics

A total of 62 children and their respective caregivers were recruited; however, 2 dyads did not answer one of the CARATKids format due to lack of time, and 9 dyad answers to eCARATKids were lost due to failure in the transfer to the secure server and were excluded. Therefore, only 51 dyads were included in the final analysis. Table 1 presents the demographic and clinical characteristics of the participants. Children’s median age was 9 years (IQR 8‐11), and 57% (29/51) were boys. Caregivers’ median age was 41 years (IQR 37-45), and 82% (42/51) were female. Children’s median school year was 4th grade (IQR 2nd–6th), and 73% (37/51) of caregivers had at least secondary education.

**Table 1.** Demographic and clinical characteristics of the participants.

Characteristics	Children (n=51)	Caregivers (n=51)
Male, n (%)	29 (57)	9 (18)
Age (years), median (IQR)	9 (8-11)	41 (37‐45)
Years of education, median (IQR)	4 (2-6)^a^	—^b^
Secondary education or higher, n (%)	—	37 (73)^a^
Smart device ownership, n (%)	45 (88)	50 (98)
Health or fitness mobile apps use, n (%)	12 (24)^a^	33 (65)

a 1 missing value.

b Not applicable.

### CARATKids Scores

The median total score was 5 (IQR 3-8) points for pCARATKids and 5 (IQR 3-7) points for eCARATKids. Disease was not controlled in 49% (25/51) of patients based on pCARATKids and in 45% (23/51) based on eCARATKids (see Table 2). A total of 24 (47%) children answered the eCARATKids first, and 4 children (8%) required assistance in reading the test in both paper and electronic versions. pCARATKids and eCARATKids were assessed with a median time interval of 41 (IQR 22-65) minutes. The time in-between assessments did not significantly influence the difference in total scores between the paper and app versions (see Multimedia Appendix 3).

**Table 2.** Control of Allergic Rhinitis and Asthma Test for children (CARATKids) scores and classification assessed on paper (pCARATKids)^a^ and on the app (eCARATKids)^b^.

Scores	pCARATKids (n=51)	eCARATKids (n=51)
Children’s subscore, median (IQR)	4 (2-5)	4 (2-5)
Caregivers’ subscore, median (IQR)	1 (0‐2)	1 (0‐2)
Total score, median (IQR)	5 (3-8)	5 (3-7)
Total score classification, n (%)		
Controlled	18 (35)	19 (37)
Insufficiently controlled	8 (16)	9 (18)
Uncontrolled	25 (49)	23 (45)

a pCARATKids: paper version of Control of Allergic Rhinitis and Asthma Test for Children.

b eCARATKids: electronic version of Control of Allergic Rhinitis and Asthma Test for Children.

### CARATKids Measurement Properties

The internal consistency of the CARATKids total score was above 0.7 in both versions (pCARATKids Cronbach *α*=0.79, 95% CI 0.56‐1.00 and eCARATKids Cronbach *α*=0.83, 95% CI 0.62‐1.00). The children’s subscore had a Cronbach α of 0.65 (95% CI 0.30‐1.00) for the paper version and 0.71 (95% CI 0.38‐1.00) for the electronic version, while the caregivers’ subscore had a Cronbach α of 0.75 (95% CI 0.32‐1.00) for the paper version and 0.78 (95% CI 0.38‐1.00) for the electronic version.

**Figure 1.**
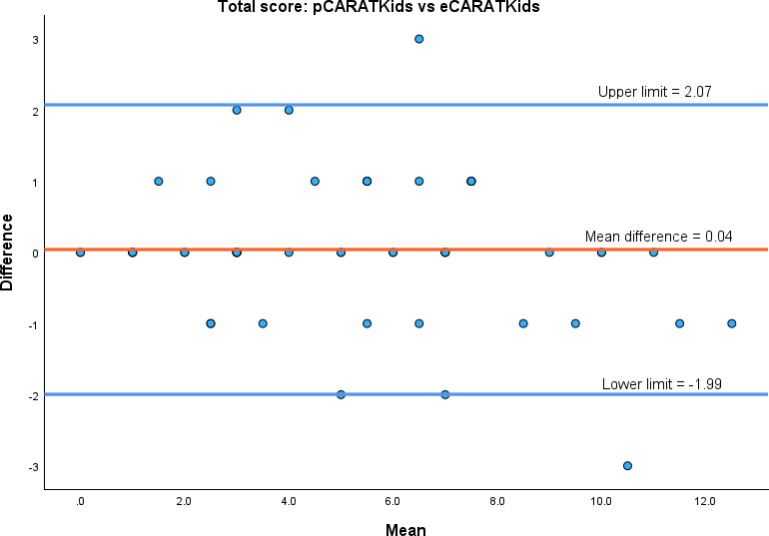
Bland-Altman plot of the total scores of Control of Allergic Rhinitis and Asthma Test for children (CARATKids), assessed on paper (pCARATKids) and on the app (eCARATKids). The blue lines represent the 95% CI.

The CARATKids demonstrated an excellent relative test-retest reliability (ICC 0.95, 95% CI 0.91‐0.97). This high reliability was maintained when CARATKids subscores were considered: children’s subscore (ICC 0.90, 95% CI 0.83‐0.94) or caregivers’ subscore (ICC 0.94, 95% CI 0.90‐0.97). The absolute reliability between the paper-based (pCARATKids) and electronic (eCARATKids) versions was good, with a mean difference between the two versions of 0.04 points and with 95% CI ranging from −1.99 to 2.07 (see Figure 1). The data points are evenly distributed around the mean difference line, with no significant proportional bias observed (see Figure 1 and Multimedia Appendix 4).

**Figure 2.**
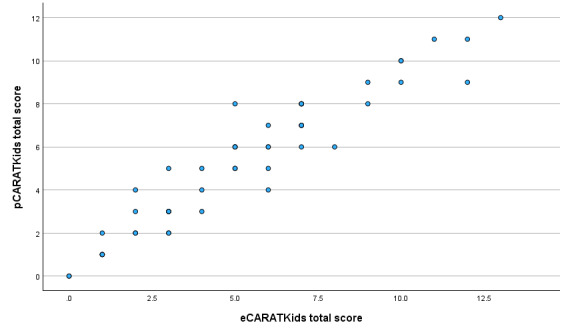
Scatter plot showing the relationship between Control of Allergic Rhinitis and Asthma Test for children (CARATKids) total score on paper (pCARATKids) and on the mobile app (eCARATKids).

Regarding convergent validity, the overall correlation between paper and mobile app scores was strong (ρ=0.95; *P*<.001; see Figure 2). When analyzed separately, both the children’s (ρ=0.89; *P*<.001) and caregivers’ (ρ=0.93; *P*<.001) subscores demonstrated strong positive correlations (see Multimedia Appendix 5).

### CARATKids Preferred Versions

Most of the children (63%, 32/51) and caregivers (61%, 31/51) reported being indifferent to the used versions, while 33% (17/51) of children and 35% (18/51) of caregivers preferred the electronic version (see Multimedia Appendix 6).

## Discussion

### Principal Findings

This study demonstrated that the electronic version of CARATKids, administered via a mobile app, is a valid and reliable alternative to the paper-based format for assessing asthma and allergic rhinitis control in children. The electronic version exhibited psychometric properties comparable to those of the traditional paper-based version, confirming its equivalence. Furthermore, the mobile app version was highly accepted by both children and caregivers, emphasizing its feasibility and user-friendliness.

### Comparison With Previous Work

The internal consistency of CARATKids was evaluated using Cronbach α, which exceeded 0.7 for the total score in both the paper and electronic versions, meeting established psychometric standards [27]. The eCARATKids version demonstrated a Cronbach α of 0.83, comparable to that of the pCARATKids version in our study (Cronbach *α*=0.79). These findings are consistent with results from other studies using paper versions, including the multicenter Portuguese version of CARATKids (Cronbach *α*=0.80), as well as the Brazilian (Cronbach *α*=0.81), Turkish (Cronbach *α*=0.84), and Dutch (Cronbach *α*=0.81‐Cronbach *α*=0.85) validations [11,12,14,15]. Furthermore, the internal consistencies of the electronic versions of CARATKids and CARAT (Cronbach *α*=0.72‐Cronbach *α*=0.81) were found to be similar [23].

The intraclass correlation coefficient (ICC 0.95, 95% CI 0.91‐0.97) further confirmed the strong test-retest reliability of the electronic version. In the validation of the mobile app’s adult version, CARAT, the ICC between the paper and electronic versions ranged from 0.65 to 0.85 [23]. The reliability of the CARATKids results was further supported by Bland-Altman, which revealed a high level of agreement between the two versions. These apparently superior results may be attributed to the shorter time interval between administrations in this study (median 41 min, IQR 22-65 min) compared to the adult app validation study, in which 85% of participants completed both the paper and electronic versions on the same day, but still with a longer interval between administrations (median 0 d, IQR 0‐2 d). Our study also revealed good convergent validity between pCARATKids and eCARATKids (ρ=0.95), indicating that both versions yield highly comparable results. In the validation of the adult mobile app version, the correlation was lower, ranging from 0.64 to 0.82 [23]. This difference may once again be explained by the shorter time interval between administrations in our study, which likely minimized variability due to changes in symptoms and increased recall bias. Furthermore, the response format may have contributed to this difference, as the children’s version is based on a yes or no format, whereas the adult version is answered on a 4-point Likert scale [11,23].

Beyond psychometric validation, the study assessed patient and caregiver acceptability. Most children (63%, 32/51) and caregivers (61%, 31/51) were indifferent to the used version, and approximately one-third preferred the electronic version, while only 4% (2/51) favored the paper version. This distribution suggests that eCARATKids does not introduce additional burden or complexity for users, as the relatively low preference for the paper version (4%) and the significant proportion favoring the electronic version (approximately one-third) indicate a notable openness to digital tools among this population. These findings align with trends observed in other pediatric studies evaluating preferences for ePROMs. For example, in orthopedic studies, 70% of participants preferred the electronic version, while in rheumatology, 83% were either indifferent or preferred the electronic version [30,31]. Furthermore, this high level of acceptability may further increase even more, considering that patients value features such as real-time progress tracking, motivational reminders, and personalized feedback [32].

### Strengths and Limitations of the Study

The evaluation of the CARATKids questionnaire’s reliability adhered to the COSMIN Risk of Bias guidelines, ensuring a methodologically robust approach [33]. Standardized measurement conditions were maintained throughout the study: both the paper-based and electronic versions were completed in the same controlled environment. Participants exclusively used the researcher’s smartphone to complete the eCARATKids, with no use of personal devices, thereby minimizing variability in response conditions. To further reduce potential bias, the researchers responsible for administering and scoring the questionnaires were blinded to previous results. In addition, participants were not informed of their total scores after completing each questionnaire, preventing any potential influence on subsequent responses. The randomized administration of pCARATKids and eCARATKids—alternating before and after appointments—was designed to mitigate order bias and any potential influence of the clinical consultation on questionnaire responses.

Despite the promising findings, this study has several limitations that warrant consideration. First, the research was conducted at a single tertiary care hospital with the minimum recommended number of participants by COSMIN, which may restrict the generalizability of the results to other health care settings, such as primary care or community-based clinics. Although the order of questionnaire administration was randomized, we did not assess if answering the electronic version first had a role on the psychometric properties obtained. The short time interval between administrations also leaves open the possibility of learning effects, which could influence responses. In addition, eCARATKids was completed on the researchers’ smartphones rather than on the participants’ personal devices. While this approach streamlined the process by avoiding installation delays and bypassing internet restrictions in the clinical environment, the use of an unfamiliar device may have introduced unintended biases or usability challenges compared to responses provided on the child’s or caregiver’s own device.

To overcome these limitations, future studies should adopt a multicenter approach aiming to include at least 100 participants, ensuring a more diverse population and broader applicability using the patient’s own device. In addition, a larger interval should separate pCARATKids and eCARATKids applications. According to COSMIN, the time interval in-between administrations should be long enough to prevent recall bias yet short enough to ensure that patients have not undergone changes in the construct being measured [27]. A time interval of about 48 hours or 1 week is often considered suitable for the evaluation of patient-reported outcomes instruments [23,34]. Future studies could benefit from the inclusion of additional reliability statistics recommended by COSMIN, such as the McDonald ω, and evaluate the responsiveness of eCARATKids over time, detecting changes in symptoms longitudinally. Future research could additionally explore the acceptability of eCARATKids among health care professionals, as this study focused primarily on children and caregivers.

### Future Directions

Given these promising findings, eCARATKids emerges as a feasible alternative to paper-based assessments, reinforcing the growing adoption of ePROMs in pediatric asthma and allergic rhinitis care. Ongoing studies are assessing ePROMs’ impact on chronic diseases, including in pediatric populations [35,36]. However, a systematic review on telemonitoring of pediatric asthma in outpatient settings identified a significant gap in the literature regarding technologies specifically designed for preschool children [37]. Further research and collaboration among healthcare providers, researchers, and technology developers are essential to advancing telemonitoring for pediatric asthma [37]. With this study, we contribute to bridging this gap.

To further improve eCARATKids, future versions of the app could include features such as biweekly reminders to encourage regular symptom tracking and improve adherence. Users could also receive immediate feedback after completing the questionnaire, including their total score and the corresponding classification of symptom control. Visual tools, such as an integrated dashboard with graphs or charts, could help caregivers track symptom changes over time, promoting better self-management. In addition, enabling users to easily share their results and symptom trends with their pediatrician would support remote consultations and personalized care. These updates could enhance patient engagement, streamline clinical decision-making, and ultimately improve asthma and allergic rhinitis management in children. Future studies could assess the impact of these features on adherence, symptom monitoring, and clinical outcomes.

### Conclusions

This study demonstrated that eCARATKids is a valid and reliable alternative to the paper-based format for assessing asthma and allergic rhinitis control in children. With strong psychometric properties and high acceptability among children and caregivers, eCARATKids has the potential to enhance telemedicine strategies and facilitate remote pediatric monitoring. Its integration into mHealth platforms could further optimize disease management and contribute to improving health outcomes in pediatric asthma and allergic rhinitis care.

## Supplementary material

10.2196/73531 Multimedia Appendix 1 Control of Allergic Rhinitis and Asthma Test for Children (CARAT-Kids) paper version: children’s questions (a); caregivers’ questions (b).

10.2196/73531 Multimedia Appendix 2 The electronic version of the Control of Allergic Rhinitis and Asthma Test for Children (eCARATKids) is available through the InspirersKids mobile app, where questions are presented one at a time. A pop-up notification alerts users when the child's section is completed, signaling the start of the caregiver's section.

10.2196/73531 Multimedia Appendix 3 Scatter plot showing the relationship between minutes between assessments and difference in Control of Allergic Rhinitis and Asthma Test for Children (CARATKids) total scores (paper - pCARATKids - and electronic versions - eCARATKids).

10.2196/73531 Multimedia Appendix 4 Bland-Altman plot of the children’s (a) and caregivers’ (b) subscores of Control of Allergic Rhinitis and Asthma Test for Children (CARATKids), assessed on paper (pCARATKids) and on mobile app (eCARATKids). The blue lines represent the 95% limits of agreement.

10.2196/73531 Multimedia Appendix 5 Scatter plot showing the relationship between Control of Allergic Rhinitis and Asthma Test for Children (CARATKids) children’s score (a), and caregivers’ score (b) on paper (pCARATKids) and on mobile app (eCARATKids).

10.2196/73531 Multimedia Appendix 6 Control of Allergic Rhinitis and Asthma Test for Children (CARATKids) version preference: mobile app version (eCARATKids), paper version (pCARATKids), or indifference between the two.

## Abbreviations

CARAT: Control of Allergic Rhinitis and Asthma Test
CARATKids: Control of Allergic Rhinitis and Asthma Test for Children
COSMIN: COnsensus-based Standards for the selection of health Measurement INstruments
eCARATKids: electronic version of Control of Allergic Rhinitis and Asthma Test for Children
ePROM: electronic patient-reported outcome measure
ICC: intraclass correlation coefficient
pCARATKids: paper version of Control of Allergic Rhinitis and Asthma Test for Children
PROM: patient-reported outcome measure
STROBE: Strengthening the Reporting of Observational Studies in Epidemiology
