# Supplementary material for: Validation and Acceptability of the Mobile App Version of the Control of Allergic Rhinitis and Asthma Test for Children (CARATKids): Cross-Sectional Study
Source: JMIR Pediatr Parent. 2025 Jul 31;8:e73531. doi: 10.2196/73531 (PMC12313081; doi:10.2196/73531)
Supplement: Multimedia Appendix 1 [file pediatrics-v8-e73531-s001.pdf]

(To be filled out by the child)

Please mark with an ☒ the answer that better describes the way you felt because of your allergic respiratory illness : asthma / rhinitis **during the last 2 weeks**.

Name: ____________________ Date: __/__/____

Because of your allergic respiratory illness : asthma / rhinitis **during the last 2 weeks**

		Yes	No
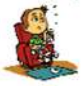	1. Have you had **stuffy nose**?	☐	☐
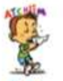	2. Have you **sneezed**?	☐	☐
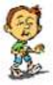	3. Have you had **runny nose**?	☐	☐
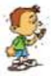	4. Have you had **shortness of breath**?	☐	☐
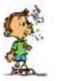	5. Have you had a **high pitch sound in chest or wheezing**?	☐	☐
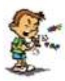	6. Have you had **cough**?	☐	☐
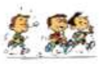	7. **During exercise or when you laugh,** have you had cough, wheezing or chest tightness?	☐	☐
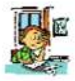	8. **Have you had tiredness/difficulty in doing your activities** because of your allergic respiratory illness : asthma / rhinitis?	☐	☐

Addition of answers Yes ☐

Now is time for parents!

(To be filled out by the parents or guardian)

Please mark with an ☒ the answer that better describes the your son /daughter felt because of the allergic respiratory illness : **asthma / rhinitis during the last 2 weeks**.

Name (son/daughter): ______________________ Age _____ Sex ___
Date: __/__/____

<u>**During the last 2 weeks**</u>, has your son /daughter

		Yes	No
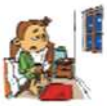	1. **Had woke up in the middle of the night** because of the allergic respiratory illness : asthma / rhinitis?	☐	☐
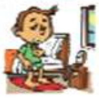	2. **Had complaints / symptoms in the morning when he/she wakes up** because of the allergic respiratory illness : asthma / rhinitis?	☐	☐
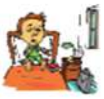	3. **Had to miss school or other activities** because of the allergic respiratory illness : asthma / rhinitis?	☐	☐
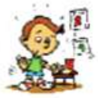	4. **Had to use / increase the use of medicines because he / she was worse of** the allergic respiratory illness : asthma / rhinitis?	☐	☐
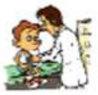	5. **Thad to go to the doctor** because he / she was worse of the allergic respiratory illness : asthma / rhinitis?	☐	☐

Addition of answers Ye (parents/guardian) ☐

Total (child+parents) ☐
