# Supplementary figures and images for: Validation and Acceptability of the Mobile App Version of the Control of Allergic Rhinitis and Asthma Test for Children (CARATKids): Cross-Sectional Study
Source: JMIR Pediatr Parent. 2025 Jul 31;8:e73531. doi: 10.2196/73531 (PMC12313081; doi:10.2196/73531)

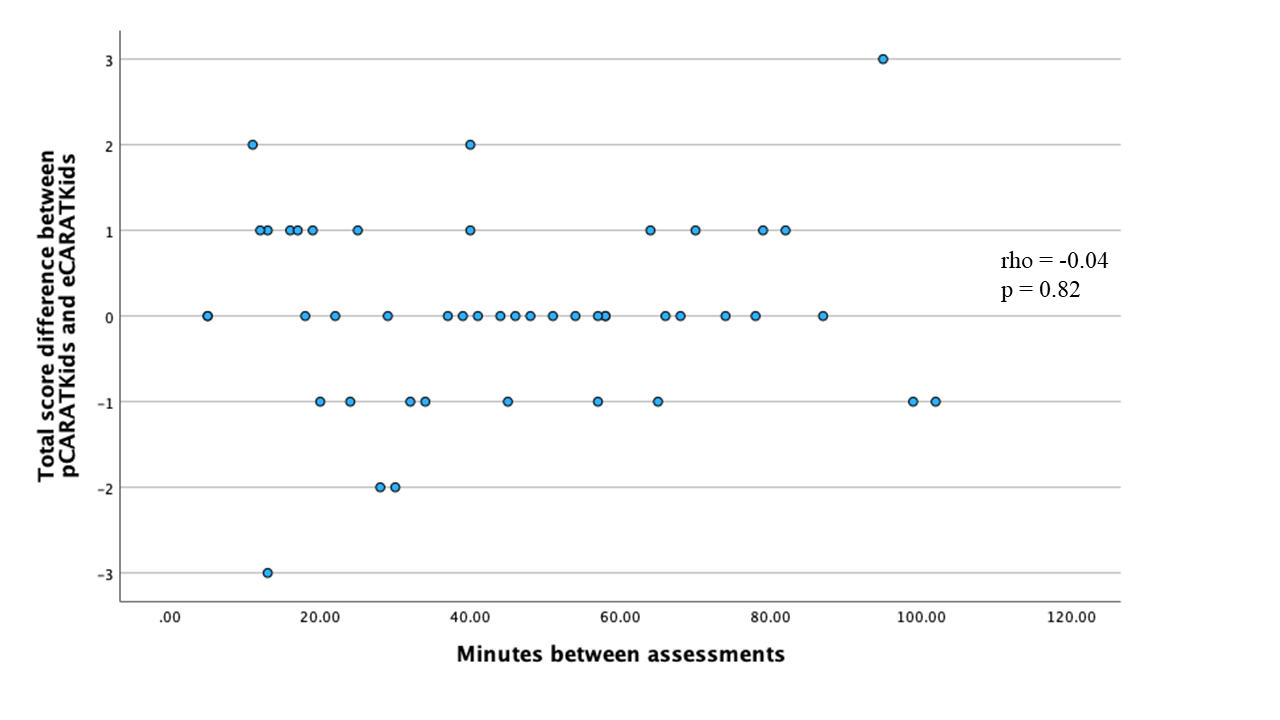

Supplement: Multimedia Appendix 3 [file pediatrics-v8-e73531-s003.png]

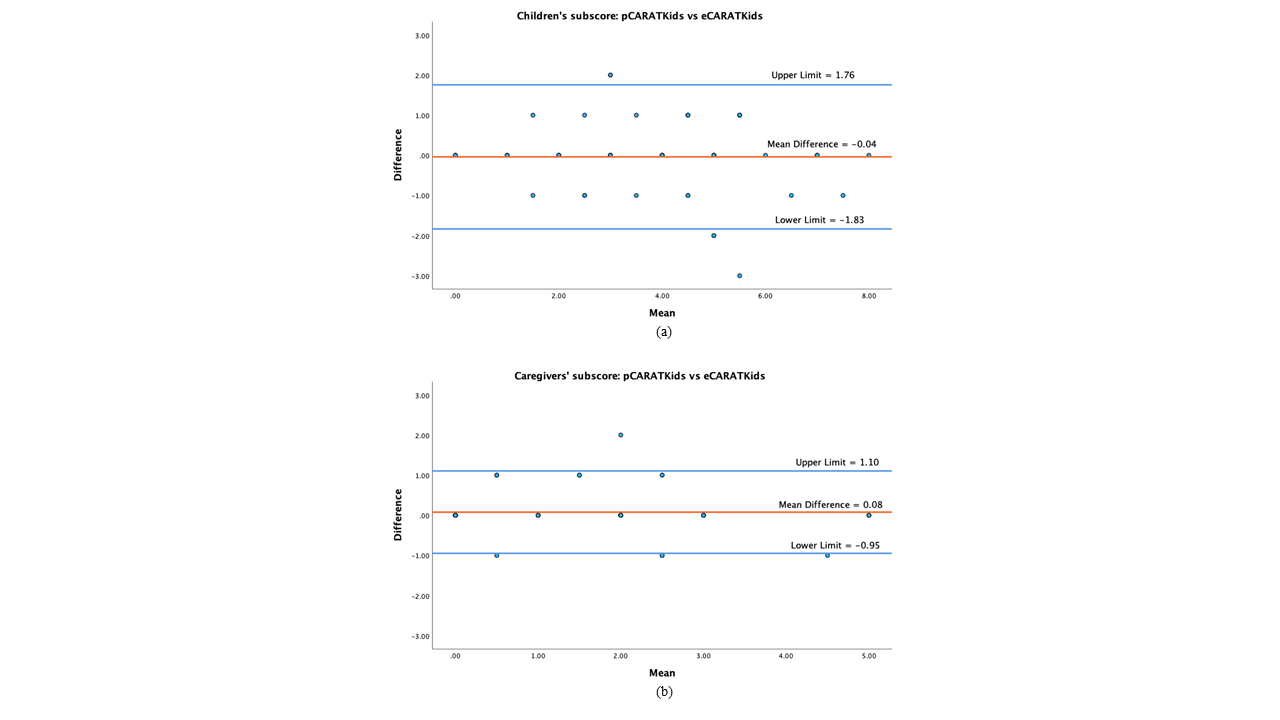

Supplement: Multimedia Appendix 4 [file pediatrics-v8-e73531-s004.png]

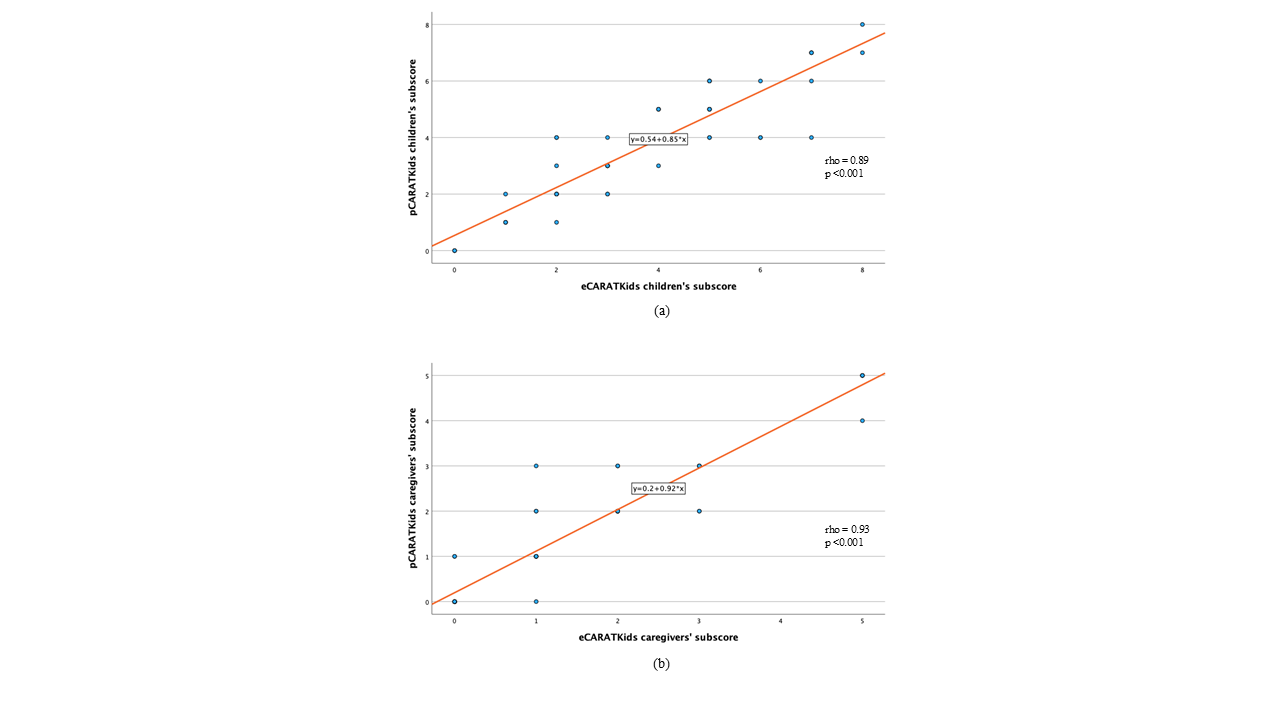

Supplement: Multimedia Appendix 5 [file pediatrics-v8-e73531-s005.png]

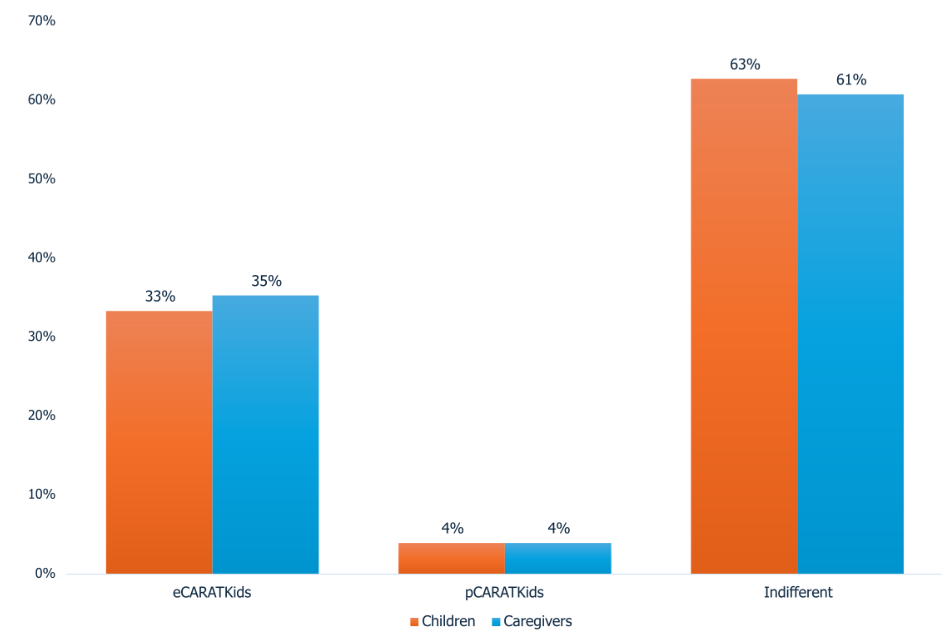

Supplement: Multimedia Appendix 6 [file pediatrics-v8-e73531-s006.png]
